# Light-curing of orthodontic bracket adhesive by transillumination through dentine and enamel

**DOI:** 10.1080/26415275.2019.1632709

**Published:** 2019-06-24

**Authors:** Erika Mäkinen, Lippo Lassila, Juha Varrela, Pekka Vallittu

**Affiliations:** a Institute of Dentistry, University of Turku, Turku, Finland;; b Turku Clinical Biomaterials Centre – TCBC, University of Turku, Turku, Finland;; c Department of Oral Development and Orthodontics, University of Turku, Turku, Finland;; d Welfare Division, University of Turku, Turku, Finland;; e Department of Biomaterials Science, University of Turku, Turku, Finland

**Keywords:** Transillumination, orthodontic bracket, polymerization

## Abstract

Bonding properties of light-curing adhesive cured by transillumination through the tooth were compared to those achieved by the conventional technique. The study analyzed the degree of cure (DC%), debonding force (DF) and adhesive remnant index (ARI) when light was transmitted through dental hard tissues.

Slices of dentin and enamel of 1 mm in thickness were combined with total thicknesses of 3 or 4 mm to simulate tooth structure without the pulp tissue. DC% with curing time of 20 s, 40 s and 60 s and irradiance power was measured for each group (*n* = 5). Brackets were bonded using transillumination on extracted incisors (*n* = 6) and premolars (*n* = 10), and DF was measured and ARI was scored.

No statistical difference was found in light transmission between the simulated samples and incisors (*p* > .05). Increasing the curing time from 40 s to 60 s enhanced the DC% only in premolars (*p* < .05). An adequate DF was achieved through transillumination both in incisors and premolars, but in premolars, the DC% remained low compared to conventionally cured brackets. Most of the bracket failures resulted from weak bracket-adhesive bond.

## Introduction

Bonding of an orthodontic bracket is based on curing of resin composite and adhesives which forms mechanical attachment between the resin and bracket material and enamel. Resin composite and adhesive are interlocked mainly by penetration of the adhesive into the microirregularities of the enamel surface and formation of so-called resin tags. Mechanical properties of the adhesive resins and resin composites, such as the flexural modulus, compressive strength and tensile strength, depend on the degree of cure (or monomer conversion) (DC%) of the adhesive resin [1–3].

Most studies that have investigated the quality of the attachment of brackets to the tooth, have used the conventional curing method with light directed from the sides of the bracket [1,4]. However, convexity of the labial tooth surface and the bracket material hinder or obstruct direct light propagation resulting in incomplete polymerization of the adhesive at the center of the bracket. We have shown previously that polymerization of the adhesive under a metal bracket can be enhanced by adding light transmitting glass fibers in the resin interface [5,6].

As an alternative method to increase the degree of cure, light curing through the tooth has been suggested [7,8]. However, there are only a few studies that have investigated the viability of this curing method [9,10]. The results of Oesterle and Shellhart [9] showed that transillumination through extracted maxillary incisors resulted in sufficiently high bond strengths of brackets, particularly when the curing time was 50 s. From the clinical point of view, the optimal bond strength has been considered to be at least 5.9 MPa [11], whereas the risk for enamel fractures increases when the bond strength is higher than 9.7 MPa [12]. Heravi et al. [10] on the other hand, found that light curing through premolars resulted in bond strengths below clinically acceptable values even if curing time was increased to 80 s. In a previous study we studied the light propagation through the dental hard tissues and showed that light penetrates enamel better than dentin, and that penetration of light is enhanced if enamel and dentin are moisturized [6].

Despite the fact that most of the manufacturers advise to cure the orthodontic adhesive from the sides of the bracket, sometimes it has some clinical difficulties. For example, when bonding brackets to the lower incisors, narrow spaces between the teeth can complicate placing the relatively large light curing tip to the sides of the bracket. Therefore, studies concerning light curing trough the tooth are important not only to orthodontics but also for prosthetic treatment, e.g. curing ceramic fillings through the enamel.

To further investigate the viability of transillumination as a light curing method in orthodontics, this study analyzed the DC% of a light-cured orthodontic adhesive, cured by transillumination through extracted incisors and premolars, and through artificial dentin-enamel systems. Furthermore, the debonding forces at the removal of the brackets were measured and a fracture analyses were carried out to locate the site of the adhesive failure.

## Material and methods

The DC% of the light curing orthodontic adhesive resin composite was measured after curing through slices of dentin and enamel or through the entire teeth. The tooth slices were prepared using a total of 60 extracted sound human third molars. The teeth, stored refrigerated in chloramine T- solution, were cut into 1 mm thick slices with a histological saw (Secotom-50, Struers A/S, Ballerup, Denmark) and finished by hand into round shape in water cooling with polishing machine (LaboPol-1, Struers A/S, Ballerup, Denmark) with a 500 grit (FEPA) SiC paper. Thickness of the slices was ensured with electronic digital caliper with accuracy of ±0.02 mm. After preparing, the slices were stored in distilled water. 40 enamel slices and 20 dentin slices were cut with histological saw vertically at bucco-lingual axis of the tooth. All the slices were then treated with 19.5% File Eze® for 1 min from both sides and then carefully rinsed with tap water. All materials used in this study are summarized in Table 1.

**Table 1.** Materials used in this study.

Material	Manufacturer	Lot no.	Content
File Eze®	Ultradent Products Inc. (505 West 10200 South Jordan, UT 84095)	BB9GB	19% EDTA
Transbond^TM^ XT Light Cure Adhesive Paste	3M Unitek (Monrovia, CA, USA)	N568393	Bis-GMA, TEGDMA
Scotchbond^TM^ Universal Etchant	3M ESPE (Deutschland, Neuss, Germany)	571374	32% Phosphoric acid
Transbond^TM^ XT Light Cure Adhesive Primer	3M Unitek (Monrovia, CA, USA)	N635136	
Ortomat Mini-Mat Standard brackets	Ortomat Herpola, Scafati, Italy	14H515H (Incisors), 15D274D (Premolars)	Stainless steel

Bis-GMA indicates bisphenol A diglycidyl ether dimethacrylate, TEGDMA triethylene glycol dimethacrylate, EDTA ethylenediaminetetraacetic acid.

For measuring the light-curing efficacy through dental hard tissues, five experimental groups of different thicknesses of dentin/enamel were created. First group (Group 1) was a control where the adhesive was directly light cured without having any solid materials between the light curing tip and the adhesive. In Groups 2 and 3, there were enamel/dentin samples of combined thickness of 3 mm and 4 mm between the light curing tip and the adhesive. In Group 2, three slices (each 1 mm in thickness) were used to create 3 mm thick sample (enamel-dentin-enamel) and in Group 3 four slices were used (enamel-dentin-dentin-enamel), respectively. Groups 2 and 3 were made to study the effect in light attenuation when the sample size is increased and to simulate natural tooth variation without the effect of the non-vital pulp tissue, present in the extracted teeth. In Group 4 an incisor and in Group 5 a premolar was placed between the light curing tip and the adhesive. Group codes are explained in Table 2.

**Table 2.** Group numbers and description for the DC% measurement.

Group number	Description	Thickness
1	control	No solid material between the light curing tip and the adhesive
2	Enamel-dentin-enamel	3 mm
3	Enamel-dentin-dentin-enamel	4 mm
4	Incisor	5.6 mm
5	Premolar	8.2 mm

For keeping the dentin/enamel slices one on the other and eliminating curing light go round the sample to the sensor of the Fourier Transform Infrared Spectrometer (FT-IR), light protecting silicone putty molds were fabricated. Molds of two heights (3.5 mm for Group 2 and 4.5 mm for Group 3) were made from Lab-Putty (Coltène/Whaledent AG, 9450 Switzerland) with round holes in the middle (Ø 6 mm) where the specimen was built in. Molds were also made individually to each extracted teeth (for Groups 4 and 5) to keep them steady on the sensor through the light curing procedure and DC% measuring. A small amount of the adhesive (Transbond^TM^ XT) was applied onto the FT-IR sensor (ZnSe-crystal, Ø 3.1 mm) followed by placing the putty mold on the adhesive. The same arrangements were repeated with the incisors and premolars. After the first scan, the adhesive was light cured through the dentin/enamel slices of teeth in the mold with a hand held light curing unit (Elipar^TM^ S10, 3 M ESPE, St. Paul, MN, USA) with an output power of 1960 mW/cm^2^. All the Groups (1–5) contained three subgroups of different curing times: 20, 40 and 60 s (in all groups *n* = 5).

Before measuring the DC%, the transmitted irradiance was measured for groups 2, 3, 4 and 5 by using MARC® -spectrometer. Data was analyzed with BlueLight**®**-program (MARC**®** Resin Calibrator, BlueLight**®** analytics inc., Halifax, Nova Scotia, Canada), and the previously introduced molds were used to inhibit light scattering. The output power of the light curing unit (LCU) was 1960 mW/cm^2^ (Elipar^TM^ S10, 3 M ESPE, St. Paul, MN, USA), when there was no solid material in between. The enamel and dentin slices were randomly selected to each slice combination in the molds, and each enamel slice was used twice: first on the top of the combination and then on the bottom. When the slice had contaminated with the adhesive resin composite, it was no longer used in the study.

The degree of conversion (DC%) was measured with Fourier Transform Infrared Spectrometry (Frontier^TM^ FT-IR, PerkinElmer®, Beaconsfield Bucks, UK) with a universal attenuated total reflectance (ATR) sampling accessory and analyzed with Spectrum^TM^ (v. 10.4.2, PerkinElmer®). The DC% was plotted against time: before curing, right after curing, 3 min, 6 min, 9 min, 12 min and 15 min.

The DC% was calculated from the aliphatic C=C peak (1638 cm^−1^) and the aromatic C=C peak (1608 cm^−1^) using Equation (1).

(1) $$\mathrm{DC}\%=\left[1-\frac{C_{\mathrm{aliphatic}}/C_{\mathrm{aromatic}}}{U_{\mathrm{aliphatic}}/U_{\mathrm{aromatic}}}\right]\times 100\%$$

C_aliphatic_ = absorption peak at 1638 cm^−1^ of the cured sample
C_aromatic_ = absorption peak at 1608 cm^−1^ of the cured sample
U_aliphatic_ = absorption peak at 1638 cm^−1^ of the uncured sample
U_aromatic_ = absorption peak at 1608 cm^−1^ of the uncured sample

In the second part of this study, bracket debonding force was studied in four groups cured either by the conventional method from the side of the bracket using incisors (Group 6, *n* = 10) and premolars (Group 8, *n* = 6) or by transillumination through incisors (Group 7, *n* = 10) and premolars (Group 9, *n* = 6). Teeth were embedded inside the acrylic cylinders so that the bracket bonding surface was perpendicular to the longitudinal axis of the cylinder. The labial surface of the teeth was etched with 32% phosphoric acid (Table 1) for 30 s, and rinsed for 15 s with oil-free tap water and then dried carefully. After etching, primer (Table 1) was applied according to the manufacturer’s instructions. The adhesive was then applied to the mesh back of the bracket and pressed firmly onto the labial surface of the teeth. Any excess adhesive was carefully removed and then light cured with hand held LCU (Elipar^TM^ S10, 3 M ESPE, St. Paul, MN, USA). In the groups 6 and 8, the adhesive was cured for 20 s from the mesial side and 20 s from the distal side of the bracket. In the groups 7 and 8, the adhesive was cured for 40 s of transillumination through the tooth. After bonding, the samples were stored in distilled water at 37 °C in the dark incubator for 24 h before measuring the debonding force.

Debonding force was studied with universal testing machine (LLOYD Instruments LR30K plus, Ametek Inc., Berwyn, US) from incisal to apical direction with a crosshead speed of 1.0 mm/min. The loading tip was positioned as close as possible to the enamel surface. The sensor used was 2500 N, and the load was recorded in newtons (N) since the influence of the surface area was being ignored. After every bracket failure a fracture analysis was made. Adhesive remnant index (ARI) was scored according to USB microscope (eScope, Oriental Inspiration Limited, Hongkong, China) right after debonding. ARI was scored as follows: 0 = no adhesive on the tooth, 1 = less than ½ of the adhesive on the tooth, 2 = more than ½ of the adhesive on the tooth, 3 = all of the adhesive on the tooth and 4 = enamel fracture.

The data was analyzed with SPSS –statistical program (IBM SPSS Statistics for Windows, Version 21.0. Armonk, NY), and the level of significance was set to 0.05. The normally distributed data was analyzed with two-way analysis on variance (ANOVA) ja Tukey’s *post hoc* test.

## Results

The irradiance of transmitted light through the slices of 3 mm and 4 mm varied from 18 to 118 mW/cm^2^ and the mean values of transmitted light for groups 2–5 are presented in Table 3. Analysis of variance revealed statistically significant differences between light transmittance between groups (*p* < .001).

**Table 3.** Light irradiance intensities and standard deviations through samples of different thicknesses.

	Mean (mW/cm²)	SD
LCU tip fully against the sensor	1958.4^A^	32.4
Group 2	91.0^B^	16.5
Group 3	22.2^C^	3.0
Group 4	37.6^C^	26.6
Group 5	6.2^D^	6.9

Different superscript letters indicate statistically significant difference (ANOVA).

**Figure 1.**
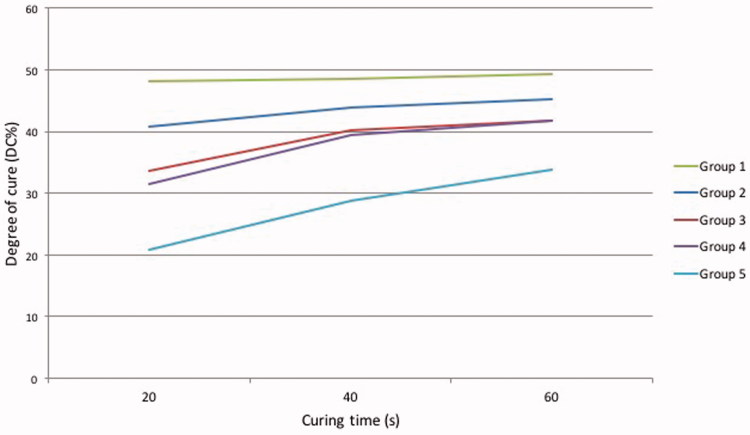
Mean degrees of monomer conversion (DC%) plotted against the curing time.

The mean values of DC% are shown in Table 4 and Figure 1. Tukey’s *post hoc* test revealed that there is no statistical difference in the DC% between groups 3 and 4 (*p* > .05). In all experimental groups DC% increased significantly when the curing time was increased from 20 s to 40 s (*p* < .05). In groups 2, 3 and 4 no significant difference in the DC% was detected between curing times of 40 s or 60 s (*p* > .05). In group 5, the difference between curing times of 40 s and 60 s was statistically significant (*p* < .005).

**Table 4.** The degree of monomer conversion (DC%) with standard deviation.

	20 s	40 s	60 s
Group 1 (Control)	48.2 (0.3) _a_^A^	48.5 (0.9) _a_^A^	49.3 (0.5) _a_^A^
Group 2	40.8 (0.9) _a_^B^	44.0 (0.9) _b_^B^	45.2 (1.9) _b_^B^
Group 3	33.7 (2.8) _a_^C^	40.2 (1.8) _b_^C^	41.8 (2.5) _b_^C^
Group 4	31.4 (7.0) _a_^C^	39.4 (2.9) _b_^C^	41.8 (2.9) _b_^C^
Group 5	20.9 (6.7) _a_^D^	28.8 (3.0) _b_^D^	33.9 (1.3) _c_^D^

Vertical superscript letters describe statistical difference between groups with same curing time (20, 40 and 60 s). Horizontal lowercase letters describe statistical differences between different curing times among the group (1–5).

**Figure 2.**
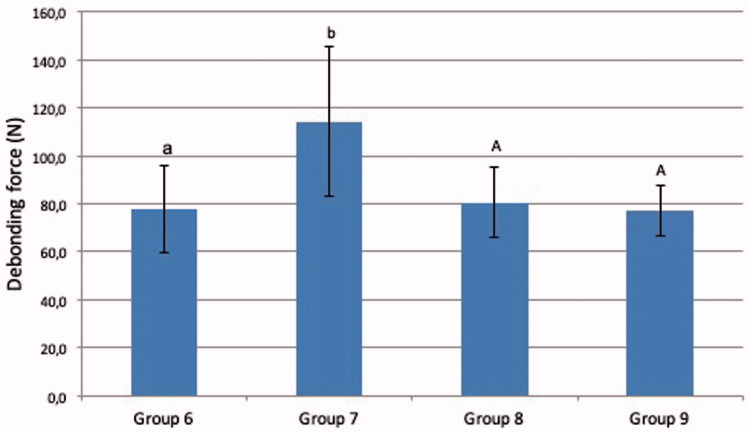
Debonding forces (N) of brackets of groups with different curing light direction. Vertical bars represent standard deviation and letters implicate statistical differences between groups 6 and 7 and between groups 8 and 9.

**Figure 3.**
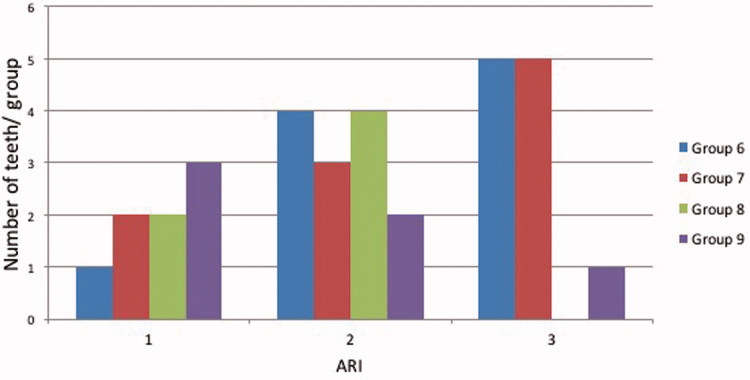
Adhesive remnant index (ARI). 0 = no adhesive on the tooth, 1 = less than ½ of the adhesive on the tooth, 2 = more than ½ of the adhesive on the tooth, 3 = all of the adhesive on the tooth and 4 = enamel fracture.

**Figure 4.**
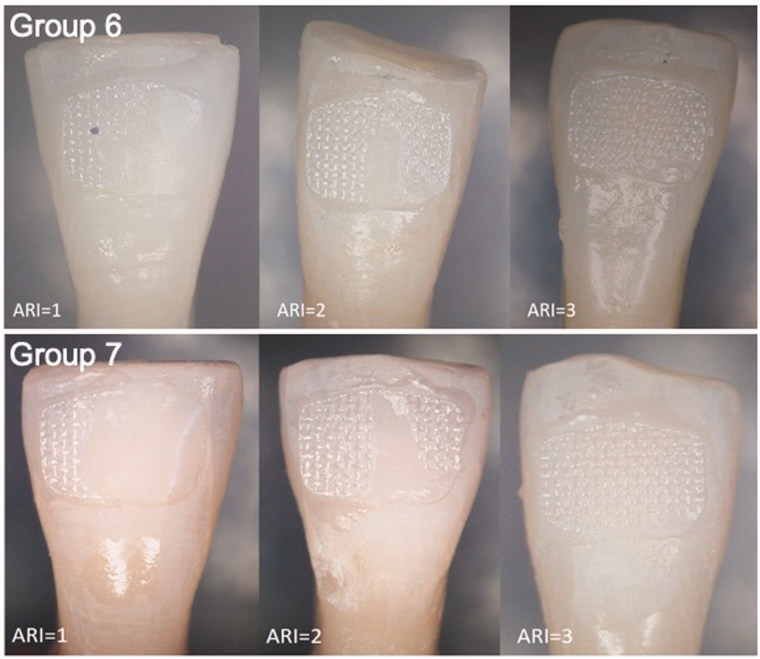
Stereomicroscope pictures right after bracket debonding. Adhesive remnant index (ARI) 0 = no adhesive on the tooth, 1 = less than ½ adhesive on the tooth, 2 = more than ½ of the adhesive on the tooth, 3 = all of the adhesive on the tooth, 4 = enamel fracture.

**Figure 5.**
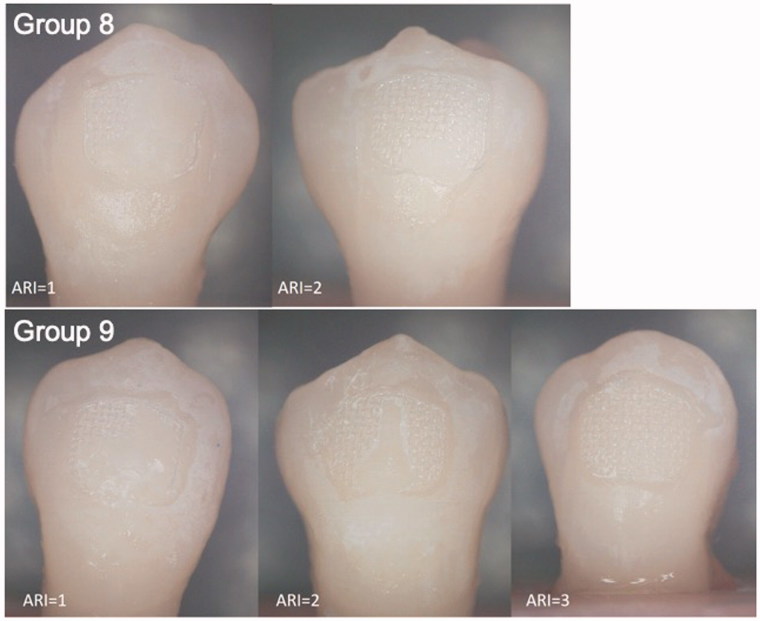
Stereomicroscope pictures right after bracket debonding. Adhesive remnant index (ARI) 0 = no adhesive on the tooth, 1 = less than ½ adhesive on the tooth, 2 = more than ½ of the adhesive on the tooth, 3 = all of the adhesive on the tooth, 4 = enamel fracture.

Bracket debonding force was 78.0 N (SD 18) in group 6 (conventionally cured incisors), 114.4 N (SD 57) in group 7 (incisors cured by transillumination), 75.1 N (SD 6) in group 8 (conventionally cured premolars) and 77.3 N (SD 10) in group 9 (premolars cured by transillumination) (Figure 2). Two-way analysis of variance and Tukey’s *post hoc* test revealed statistically significant difference between groups 6 and 7 (*p* < .05) The difference between groups 8 and 9 was not statistically significant (*p* > .05). ARIs are presented in Figures 3, 4 and 5. In 48.1% of the cases, over ½ of the adhesive remained on the tooth surface, and in 40.1% of cases, the adhesive remained entirely on the tooth surface. In no case was the adhesive completely removes with the bracket.

## Discussion

The present findings suggested that effectiveness of light curing by transillumination through layers of dental hard tissues can be close to that achieved with the conventional curing technique where light is directed from the side of the bracket. The degree of conversion after curing by transillumination through dentin/enamel layers of 3 or 4 mm, or through intact incisors varied from 31% to 45%, which is close to 35% to 45% reported in studies that investigated the effectiveness of the conventional method [1,5]. However, light curing through premolars resulted in considerably lower DC% values.

In the present study, curing the adhesive with transillumination through the premolars showed comparable bond strength values to conventionally bonded brackets. Curing light propagates through enamel by scattering along enamel rods and hydroxyapatite crystals, and at the interface between enamel and dentin, light scatters due to different refractive indexes [13]. Relatively high DF in premolars might be due to light scattering from the edges of the tooth during transillumination allowing the curing light to reach the adhesive under the bracket. It can be assumed, that the degree of cure and debonding force would have been lower with optical contact during curing. However, scattering cannot be considered as an independent phenomenon since light propagation is composed of absorbed, transmitted and reflected light [14]. Our results are thus in line with the earlier conclusions that transillumination seems to be clinically viable curing method for bonding brackets to incisors but not to premolars [9,10]. The present *in vitro* study design does not allow analysis of the light scattering effects of the pulpal tissues that has been shown to exceed that of enamel and dentin [15]. Thus, the DC% *in vivo* would be less than the present results indicate.

It has been suggested that increasing the transillumination time might result higher DC% and sufficient bond strength of the bracket [8–10]. We tested the effect of the transillumination time by increasing the curing time from 20 through 40 s to 60 s. The results showed that the DC% did increase as the curing time became longer. Due to these finding and established clinical practice the curing time in this study was set to be 40 s for both conventional method and transillumination. However, the relation of the DC% and the curing time is not linear, as the free radicals that initiate the polymerization of the adhesive become mobility limited as the DC% increases [16]. With a curing time of 60 s all test groups, including the premolars, showed conversion percentages that were within the range of the conventional curing method. However, longer curing times should be used with caution because long irradiation times, such as 60 s, can result in rise of temperature in the pulp chamber and irreversible damage of the pulpal tissues. It has been found that the temperature rise in the pulp chamber is dependent on the power density of the light curing device. With modern LED curing units the temperature rise in the pulp chamber varies from 1.2 °C to 9.4 °C [17].

In a previous study we found that the mean irradiance of transmitted light through incisors was 37.6 mW/cm^2^ and that through premolars 6.2 mW/cm^2^ [6]. Light propagation through the tooth seems to follow Beer-Lambert law, which is the relation between material absorbance and concentration. In case of human tooth, the relation is not linear due to light scattering from the dental hard and soft tissues. Therefore, we used the dentin and enamel slices in this study to exclude the influence of dissolved pulpal tissue to the light transmission through the tooth. The results of Kumar et al. [15] indicated that the level of light transmission through 13 mm thick premolar was even lower (1.08 mW/cm^2^). In spite of the lower level of light transmission through premolars, the bond strengths obtained by the transillumination method were at a clinically acceptable level, not far from those achieved by the conventional labial curing [6,15].

The adhesive remnant index (ARI) that was recorded after each bracket failure showed that the adhesive remained entirely on the tooth surface in almost 50% of the cases. In 40% of the cases over half of the adhesive remained on the tooth surface. According to these findings, it can be assumed that the bond between the adhesive and enamel is stronger than the bond between the adhesive and the metal bracket used in the study. It has been shown that the bracket base design influences to bracket bond strength. According to Wang et al., metal brackets with relatively large mesh-type bases or retention grooves provide the highest bond strengths [18]. Bonding of resin based materials to the surface of metals can be improved by silane coupling agents which are commonly used with prosthodontic devices and several other technical fields [19,20]. However, by increasing the overall bond strength of the bracket to the enamel too much, the removal of the bracket is more time consuming and potentially cause risk of enamel damage.

## Conclusions

- Bonding of orthodontics brackets by transillumination method results comparable DC% values to the conventional light curing technique with incisors but not with premolars
- Debonding forces after transillumination were adequate both in incisors and premolars
- Most of the bracket debondings in this study resulted from weak bracket-adhesive bond strength

## Data Availability

All data collected and analyzed for this study are included and presented in the published article.
